# KLF11 is an independent negative prognostic factor for breast cancer from a cohort study and induces proliferation and inhibits apoptosis in vitro

**DOI:** 10.1007/s12282-023-01470-5

**Published:** 2023-05-18

**Authors:** Lili Lin, Kristina Pfender, Nina Ditsch, Christina Kuhn, Martina Rahmeh, Lin Peng, Elisa Schmoeckel, Doris Mayr, Fabian Trillsch, Sven Mahner, Mirjana Kessler, Udo Jeschke, Anna Hester

**Affiliations:** 1 Department of Obstetrics and Gynecology, University Hospital, LMU Munich, Marchioninistr. 15, 81377 Munich, Germany; 2 Department of Pathology, Ludwig-Maximilians University of Munich, 81337 Munich, Germany; 3 Department of Gynecology and Obstetrics, University Hospital Augsburg, 86156 Augsburg, Germany

**Keywords:** KLF11, Breast cancer, Prognostic factor, proliferation, Apoptosis

## Abstract

**Background:**

The therapy concepts that target several members of krüppel like factor (KLF) family have been achieved in breast cancer (BC). However, the role of KLF11 in BC remains unclear. This study explored the prognostic significance of KLF11 in BC patients and investigated its functional roles in this malignancy.

**Methods:**

Immunohistochemistry (IHC) staining of KLF11 in 298 patients’ samples was performed to determine the prognostic role of the KLF11. Then the protein level was correlated to clinicopathological characteristics and survival outcomes. Afterward, the function of KLF11 was explored in vitro with siRNA-mediated loss-of-function of cell viability, proliferation, and apoptosis.

**Results:**

From the cohort study, we found that the expression of KLF11 was positively associated with highly proliferative BC of BC. Furthermore, prognostic analysis demonstrated that KLF11 was an independent negative factor for disease-free survival (DFS) and distant-metastasis-free survival (DMFS) of BC. The KLF11-related prognostic model for DFS and DMFS showed high accuracy in predicting the 3-,5- and 10 -year survival probability of BC patients. Additionally, the knockdown of KLF11 inhibited cell viability and proliferation, as well as induced cell apoptosis in MCF7 and MDA-MB-231 cells, while only inhibited cell viability and induced cell apoptosis in SK-BR-3 cells.

**Conclusions:**

Our study indicated that targeting KLF11 is an interesting therapeutic concept and further research could lead to a new therapeutic improvement in BC, especially in highly aggressive molecular subtypes.

## Introduction

Breast cancer (BC) is a heterogeneous disease characterized by diverse molecular subtypes [1, 2]. BC subtypes are classified according to the expression of estrogen receptor (ER), progesterone receptor (PR), human epidermal growth factor receptor 2 (HER2), and the proliferation marker Ki-67 [3]. In recent decades, specific therapies based on these molecules have dramatically improved the prognosis of specific patient groups [4, 5, 6, 7]. However, in BC patients with ER-positive, endocrine therapies show unsatisfactory effects in about 20% of all patients due to primary or acquired resistance [8]. In addition, despite the substantial efficacy of the HER2-targeted therapies, some BC patients with HER2-amplified show inadequate responses to the treatment [9]. Furthermore, triple-negative BC (TNBC), which is defined by the absence of ER, PR, and HER2 expression, lacks similar specific targeted therapies [10]. Non-specific chemotherapy remains the primary treatment option for this subtype, indicating the urgent requirement of alternative therapeutic targets. In summary, molecular subtype classification has achieved therapy improvement. However, the heterogeneity of tumor cells enables different types of BC to have distinct therapy strategies and exhibit different therapeutic effects. In search of therapeutic improvement, exploring alternative and/or synergistic therapeutic targets for conventional targets is a promising concept for improving treatment of BC.

Krüppel like factors (KLFs) are transcriptional factors that belong to zinc finger family. Aberrant expression of KLFs is observed in BC [11]. They regulate cell proliferation, apoptosis, differentiation, invasion, migration, and cell metabolism of BC [11]. Furthermore, in different molecular subtypes of BC cells, KLFs are involved in different biological processes [11]. In addition, KLFs are highly correlated to the clinicopathological characteristics and survival outcomes of BC patients [11]. Most importantly, the therapy concepts that target KLF family have been achieved in BC[11]. As a member of the KLFs family, KLF11 regulates gene transcriptions as both an activator and a repressor [12, 13, 14]. The role of KLF11 in several cancer types is mainly growth-related, such as cell proliferation or cell apoptosis [15]. However, the prognostic relevance of KLF11 in BC patients and the cellular functions that KLF11 involved remain unclear. Therefore, it is worth exploring whether KLF11 actually acts as an oncogene or a tumor suppressor in BC, and then implying its potential ability to be a therapeutic target for BC patients.

Here, we explored the prognostic role of the KLF11 by performing immunohistochemistry (IHC) staining of KLF11 in 298 samples of BC patients, followed by clinicopathological correlation and survival analysis to assess the clinical significance of KLF11. In addition, to reveal its regulation in cellular function, we explored the siRNA-mediated loss-of-function of KLF11 by performing cell viability, cell proliferation, and cell apoptosis assays in luminal, HER2-amplified subtype, and TNBC cell lines, respectively.

## Materials and methods

### Patients

In this study, 320 consecutive patients who underwent surgery for BC from 2000 to 2002 at the Department of Obstetrics and Gynecology, Ludwig-Maximillian's-University of Munich, Germany. In the further analyses, only cases with a diagnosis of sporadic BC and without family history for BC were included (*n* = 306). Patients with primary distant metastases (*n* = 6) and patients only with ductal carcinoma in situ (DCIS) (*n* = 2) were excluded. In total, 298 patients were included. The Institute of Pathology assigned the tumor grading (according to the Elston-Ellis system). Patient data regarding patient age, ER status, PR status, HER2-amplification, metastasis, local recurrence, progression, and survival were retrieved from the Munich Cancer Registry. The surrogate molecular subtypes were defined: Luminal A-like (ER/PR + , HER2 unamplified, Ki-67 ≤ 14%), Luminal B-like (ER/PR positive, HER2 unamplified, Ki-67 > 14%), TNBC (ER-, PR-, HER2 unamplified), HER2 amplified Luminal-like (HER2 amplified, ER/PR +) and HER2 amplified non-Luminal-like (HER2 amplified, ER-, PR-) [16]. Survival outcomes were statistically analyzed after an observation period of up to 12 years.

### Ethics approval and consent to participate

This study has been approved by the Ethics Committee of the Ludwig-Maximilian-University Munich (approval number 048–08). The BC specimens were obtained in clinically indicated surgeries. All diagnostic procedures were completed when the current study was performed, and the patients' data were anonymized. The ethical principles adopted in the Declaration of Helsinki 1975 have been respected. As per declaration of our ethics committee, no written informed consent of the participants or permission to publish is needed given the circumstances described above. Researchers were blinded from patient data during experimental and statistical analysis.

### Immunohistochemistry

IHC was performed as previously described [17]. Briefly, formalin-fixed and paraffin-embedded (FFPE) slides of BC patients were used. IHC was performed using ZytoChem Plus HRP Polymer Bulk kit (Nr.POLHRP-100, Zytomed System). The samples were dewaxed. Subsequent gradually rehydrated, followed by cooking with sodium citrate buffer (pH 6.0). After blocking with Reagent 1 of the kit, the slides were incubated with the primary anti-KLF11 (1:200, Nr.H00008462-M03, Novus Biologicals) for 16 h at 4 °C. Next, the slides were post blocked with Reagent 2, followed by incubating with Reagent 3. The color was then developed by Liquid DAB + Substrate Chromogen System kit (Nr. K3468, Dako). Finally, the samples were counterstained with Mayer's hemalum and mounted. Placenta tissue was used as negative and positive control. The slides were assessed by Leitz microscope (Type 307–148.001 514686, Wetzlar).

### Evaluation of KLF11 Immunoreactive

Immunoreactive score (IRS) was calculated to evaluate the IHC result of KLF11. The IRS of KLF11 was assessed by two experienced investigators independently. Receiver operating characteristic (ROC) analysis was used to define optimal cutoff points [18]. The IRS of KLF11 ≤ 2 was categorized as "KLF11-low", and IRS > 2 was categorized as "KLF11-high". The results were then used to perform correlation analysis with clinicopathological characteristics and survival analysis using overall survival (OS), disease-free survival (DFS), distant-metastasis-free survival (DMFS), and local recurrence-free survival (LRFS) of BC as the endpoints in our study.

### Forest plots for subgroup survival analyses

Forest plots were used to display the prognostic role of KLF11 in BC subgroups grouped by clinicopathological parameters. The R package "ggplot2" was used to plot the analyzed univariate cox results. The R scripts were performed in R programmer version 3.6.3.

### Nomogram construction and calibration

Nomograms were modeled and developed using R packages "survival" and "rms". The concordance index (c-index) represents the nomogram capability of discrimination. The closer c-index is to 1.0, the higher the discriminative performance level [19]. The prediction accuracy of the established model was evaluated with a calibration curve.

### BC cell lines culture

MCF7 (Nr.86012803, ECACC), MDA-MB-231 (Nr.92020424, ECACC), and SK-BR-3 (Nr.ACC736 DSMZ) were used to explore the functional role of KLF11. The cell lines were cultured in RPMI 1640 (Nr. 61870–010, Gibco) supplemented with 10% fetal bovine serum (Nr.10270–106, Gibco). No antibiotics or antimycotics were added. All cells were incubated in 5% CO2 at 37 °C. In subsequent experiments, only cells detected to be free of mycoplasma infection were used. Neubauer cell chambers were used to count cells.

### siRNA-mediated knockdown

Firstly, BC cell lines were pre-seeded in six-well plates. siRNA-transfection was performed when the cell density reached approximately 60%. The transfection procedure was performed according to the manufacturer's protocol of the Lipofectamine RNAiMAX reagent (Nr.13778–075, Invitrogen). BC cell lines were transfected with AllStars Negative Control (NC) siRNA (Nr.1027280, Qiagen), and two independent siRNAs target KLF11: *KLF11*-S1: CACGTAGATAACCGAGAGAAT (Nr.SI04139751, Qiagen), *KLF11*-S2: AGGAAGCGGCATGACAGCGAA (Nr.SI4291175, Qiagen) and *KLF11*-S3: TTGCCGGAAGACCTACTTCAA (Nr. SI04198418, Qiagen). After 48 h, BC cell lines were harvested for protein or RNA extraction and detection or for further cell functional experiments.

### RNA extraction and real-time PCR

RNA was extracted following the manual of RNeasy Mini Kit (Nr.74104, Qiagen). Subsequent reverse transcription was carried out with Biozym cDNA Synthesis Kit (Nr.331470L, Biozym) and implemented as the manual. Real-time PCR (rtPCR) was performed to validate the knockdown of *KLF11* from mRNA level with FastStart Essential DNA Probes Master kit (Nr.06402682001, Roche) and gene-specific primers using the LightCycler Nano (Roche). *ACTB* (β-actin) was used as the reference gene. *KLF11* primers (Forward: 5’-CTTCCATTCTTTATCGACTCTGTG-3' and Reverse: 5'- GATGGCTCCACGAGATCAG-3', Nr.100154265, Roche) and *ACTB* primers (Forward: 5'- TCCTCCCTGGAGAAGAGCTA-3' and Reverse: 5'- CGTGGATGCCACAGGACT-3', Nr.100143492, Roche) were used.

### Colorimetric cell-based KLF11 ELISA

The colorimetric cell-based KLF11 ELISA Kit (Dr.DEIA-XYA1113, CD Creative Diagnostic) was used to validate the siRNA-mediated knockdown of KLF11 from protein levels. Firstly, the siRNA transfection for KLF11 knockdown was conducted as described above. Afterward, the measurement procedure and data normalization were performed as the manufactural’s manual. The absorbance of optical density (OD)_450_ and OD_595_ was measured. Three replicates were performed with each cell line. To guarantee the reliability, we repeated the experiment at least three times.

### Cell functional assays

Firstly, the siRNA transfection for KLF11 knockdown in BC cell lines was conducted as described above. After 48 h, BC cell lines were harvested for further cell functional experiments.

*Cell viability* was evaluated using the Methylthiazolyldiphenyl-tetrazolium bromide (MTT) assay. Harvested cells were then seeded (3500 cells/well) into three sets of 96-well plates with five replicates for each cell line and cultured in 10% FBS-containing RPMI 1640 medium for 24 h, 48 h, and 72 h, respectively. Then incubation ended at three different time points, and at each time point to each well, MTT solution (20 µl/well, 5 mg/ml, Nr.M5655, Sigma-Aldrich) was pipetted. After removing the MTT-containing medium, dimethyl sulfoxide (DMSO, 200 µl/well) was added. The absorbance of OD_595_ was measured.

*Cell proliferation rate* was determined using Cell Proliferation ELISA kit (Nr.11647229001, Roche). Cells were then seeded (5000 cells/well) into two sets of 96-well plates with five replicates for each cell line and cultured in10% FBS-containing RPMI 1640 medium for 24 h and 48 h, respectively. Then the entire procedure was performed according to the manual. Briefly, when the incubation ended at two different time points, supplemented each time point to each well with BrdU (20 μl/well). The absorbance of OD_450_ was measured.

*Cell apoptosis* was detected using the Cell Death Detection ELISA kit (Nr.11544675001, Roche). BC cell lines were then harvested and prepared as sample lyses for further detection. The assay was performed according to the manufacturer’s instructions. The absorbance of OD_405_ was measured.

All the absorbance was measured using an Elx800 universal Microplate Reader and analyzed using Gene 5 software. To guarantee the reliability, all the experiment was repeated at least three times.

### Statistical analysis

IBM SPSS (version 26) and Graphpad Prism 8.1 was used for statistical analysis and illustrations. Data was first subjected to normality and equal variances test. Student's t, Welch's, and Mann–Whitney *U* test were used for two-group comparisons. One-way ANOVA and Kruskal–Wallis test were used for multiple-group comparisons. Dunn's test was implemented for pairwise comparisons within the multiple groups. In addition, Chi-Square Test was performed for the comparison of categorical variables. Kaplan–Meier (KM) survival analysis was performed with Log-rank test. Univariate and multivariate Cox regression analyses were also performed for the survival analysis with a hazard ratio (HR) and a 95% confidence interval (CI). The proportional hazards assumption test was performed for each variable in all cox models using the Schoenfeld statistical test. All reported p values are two-sided. *P*-values < 0.05 were considered statistically significant.

## Results

### The expression of KLF11 was positively associated with highly proliferative BC

**Fig. 1:**
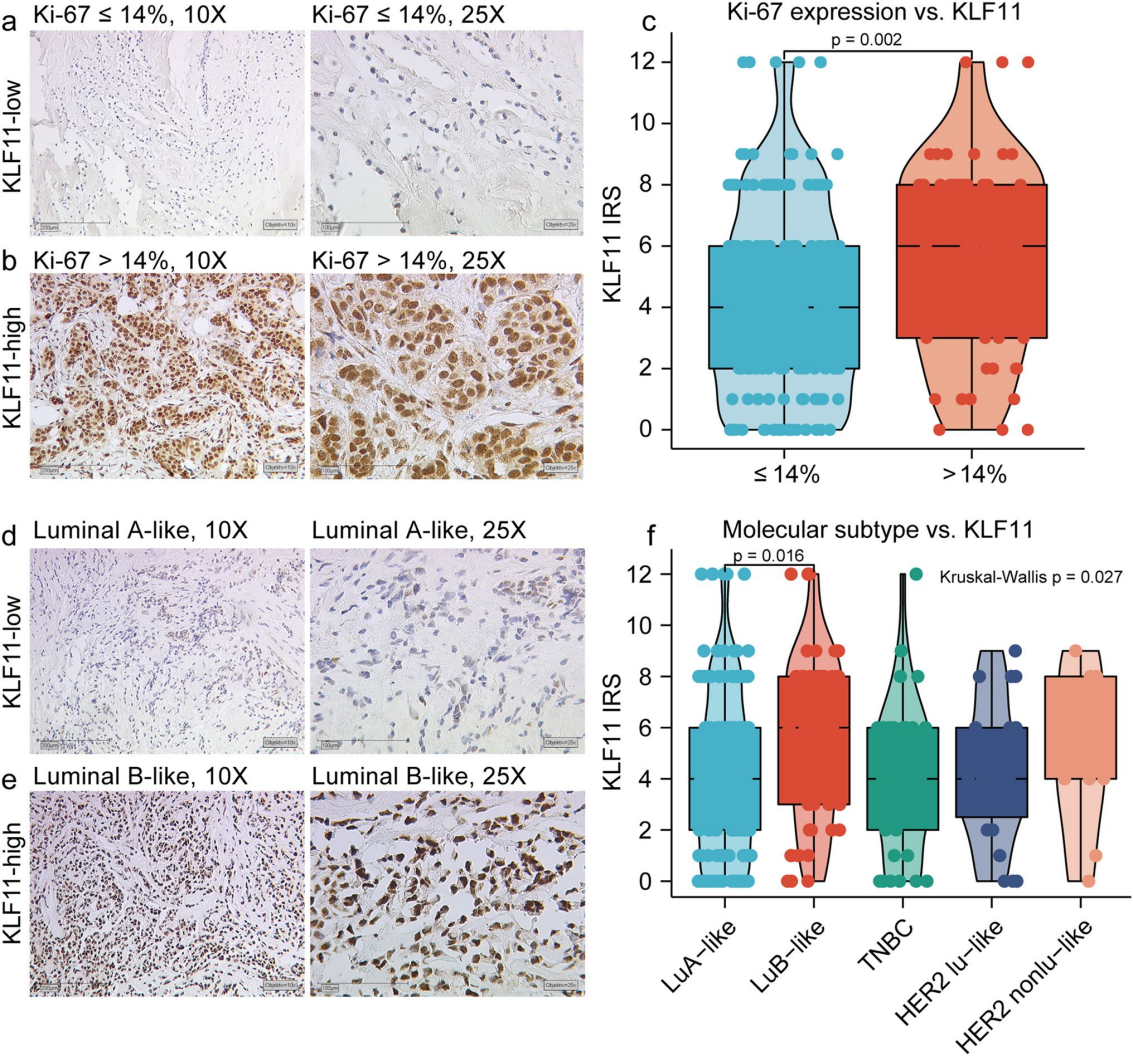
The expression of KLF11 was higher in more aggressive subtypes of BC. **a–b.** Representative Immunohistochemistry (IHC) images of KLF11 staining in BC tissues with the expression of Ki-67 ≤ 14% (**a**) and Ki-67 > 14% (**b**). Magnification: 10 X (left), 25X (right). **c.** Boxplot showed that KLF11 protein level in tumors with the expression of Ki-67 ≤ 14% is significantly lower than in tissue with Ki-67 > 14% (Mann–Whitney *U* test *p* = 0.002). **d–e** Representative IHC images of KLF11 staining in Luminal A-like (**d**) and Luminal B-like BC (**e**). Magnification: 10 X (left), 25X (right). **f** The boxplot graph shows that KLF11 was differentially expressed across the five BC subtypes (Kruskal–Wallis test *p* = 0.027). The further pairwise comparison demonstrated that the expression of KLF11 was higher in Luminal B-like BC compared to in Luminal A-like BC (Dunn's test *p* = 0.016). BC, Breast cancer; KLF11, Krüppel like Factor 11; LuA, Luminal A; LuB, Luminal B; TNBC, Triple-negative BC; HER2 Lu-like, HER2 amplified luminal-like; HER2 non-Lu-like, HER2 amplified non-luminal-like; IRS, Immunoreactive score

KLF11 was successfully stained in 292/298 samples (6 samples without sufficient staining due to technical issues). The expression of KLF11 was higher in more proliferative BC that with the expression of Ki-67 > 14% (*P* = 0.002, Fig. 1a–c). Furthermore, KLF11 was differentially expressed in different molecular subtypes of BC (*P* = 0.027, Fig. 1d-1f). The lowest KLF11 expression was observed in luminal A-like BC (Fig. 1f). The further pairwise comparison demonstrated that the expression of KLF11 was higher in Luminal B-like than in Luminal A-like BC (*P* = 0.016, Fig. 1 f). These results indicates that the expression of KLF11 was positively associated with highly proliferative BC.

In addition, of all 292 stained samples, 202 (69.2%) cases were categorized as “KLF11-high” subgroup, while 90 cases (30.8%) were characterized as “KLF11-low” subgroup. We found that the BC patients with KLF11-high expression were more in non-luminal-A than in luminal A subgroup (76.4% vs. 64.4%, *p* = 0.028, Table 1). Additionally, more BC patients with KLF11-high expression was observed in Ki-67 > 14% cases than in Ki-67 ≤ 14% cases (79.7% vs. 64.6%, *p* = 0.033, Table 1). These results indicated that the distribution of KLF11-high expression was more frequently in more aggressive BC.

**Table 1:** Distribution of KLF11 compared to clinicopathelogical parameters in the BC cohort

Characteristics	Total	KLF11-low expression	KLF11-high expression	Chi-Square Test *p* value
	Number of cases (%)	Number of cases (%)	Number of cases (%)	
*N*	292 (98.3%)	90 (30.8%)	202 (69.2%)	
Age(yo)				0.127
< 50 yo	72 (24.7%)	17 (23.6%)	55 (76.4%)	
≥ 50 yo	220 (75.3%)	73 (33.2%)	147 (66.8%)	
Tumor histology				0.995
Invasive Ductal	118 (40.5%)	36 (30.5%)	82 (69.5%)	
Invasive lobular	83 (28.5%)	26 (31.3%)	57 (68.7%)	
Mixed type	37 (12.7%)	11 (29.7%)	26 (70.3%)	
Other types	53 (18.2%)	17 (32.1%)	36 (67.9%)	
Molecular subtype				0.254
Luminal A-like	163 (56.2%)	58 (35.6%)	105 (64.4%)	
Luminal B-like	58 (20%)	12 (20.7%)	46 (79.3%)	
Triple negative	37 (12.8%)	10 (27.0%)	27 (73.0%)	
HER2 amplified luminal-like	23 (7.9%)	6 (26.1%)	17 (73.9%)	
HER2 amplified non luminal-like	9 (3.1%)	2 (22.2%)	7 (77.8%)	
Luminal A like subtype				0.028*
No	127 (43.8%)	30 (23.6%)	97 (76.4%)	
Yes	163 (56.2%)	58 (35.6%)	105 (64.4%)	
Tumor grade				0.328
G1	15 (9.3%)	6 (40.0%)	9 (60.0%)	
G2	102 (63.4%)	27 (26.5%)	75 (73.5%)	
G3	44 (27.3%)	16 (36.4%)	28 (63.6%)	
Tumor focus				0.429
Unifocal	157 (53.8%)	52 (33.1%)	105 (66.9%)	
Multifocal&Multicentric	135 (46.2%)	38 (28.1%)	97 (71.9%)	
Axillary lymph node metastasis				0.706
No	161 (56.3%)	51 (31.7%)	110 (68.3%)	
Yes	125 (43.7%)	37 (29.6%)	88 (70.4%)	
pT classification				0.907
pT1	190 (65.1%)	59 (31.1%)	131 (68.9%)	
pT2-4	102 (34.9%)	26 (30.4%)	60 (69.6%)	
ER status				0.467
Negative	56 (19.2%)	15 (26.8%)	41 (73.2%)	
Positive	236 (80.8%)	75 (31.8%)	161 (68.2%)	
PR status				0.294
Negative	123 (42.1%)	42 (34.1%)	81 (65.9%)	
Positive	169 (57.9%)	48 (28.4%)	121 (71.6%)	
HER2 status				0.467
Negative	259 (89%)	81 (31.3%)	178 (68.7%)	
Positive	32 (11%)	8 (30.6%)	24 (69.4%)	
Expression of Ki-67				0.033*
≤ 14%	164 (73.5%)	58 (35.4%)	106 (64.6%)	
> 14%	59 (26.5%)	12 (20.3%)	47 (79.7%)	

Values in bold are statistically significant

*yo* years old, *pT* pathological Tumor size, *ER* Estrogen receptor, *PR* Progesterone receptor, *HER2* Human epidermal growth factor receptor 2, Chi-Square Test was performed in this table. *p < 0.05; **p < 0.01; ***p < 0.001

### KLF11 was negatively associated with DFS, DMFS and LRFS in BC

**Fig. 2:**
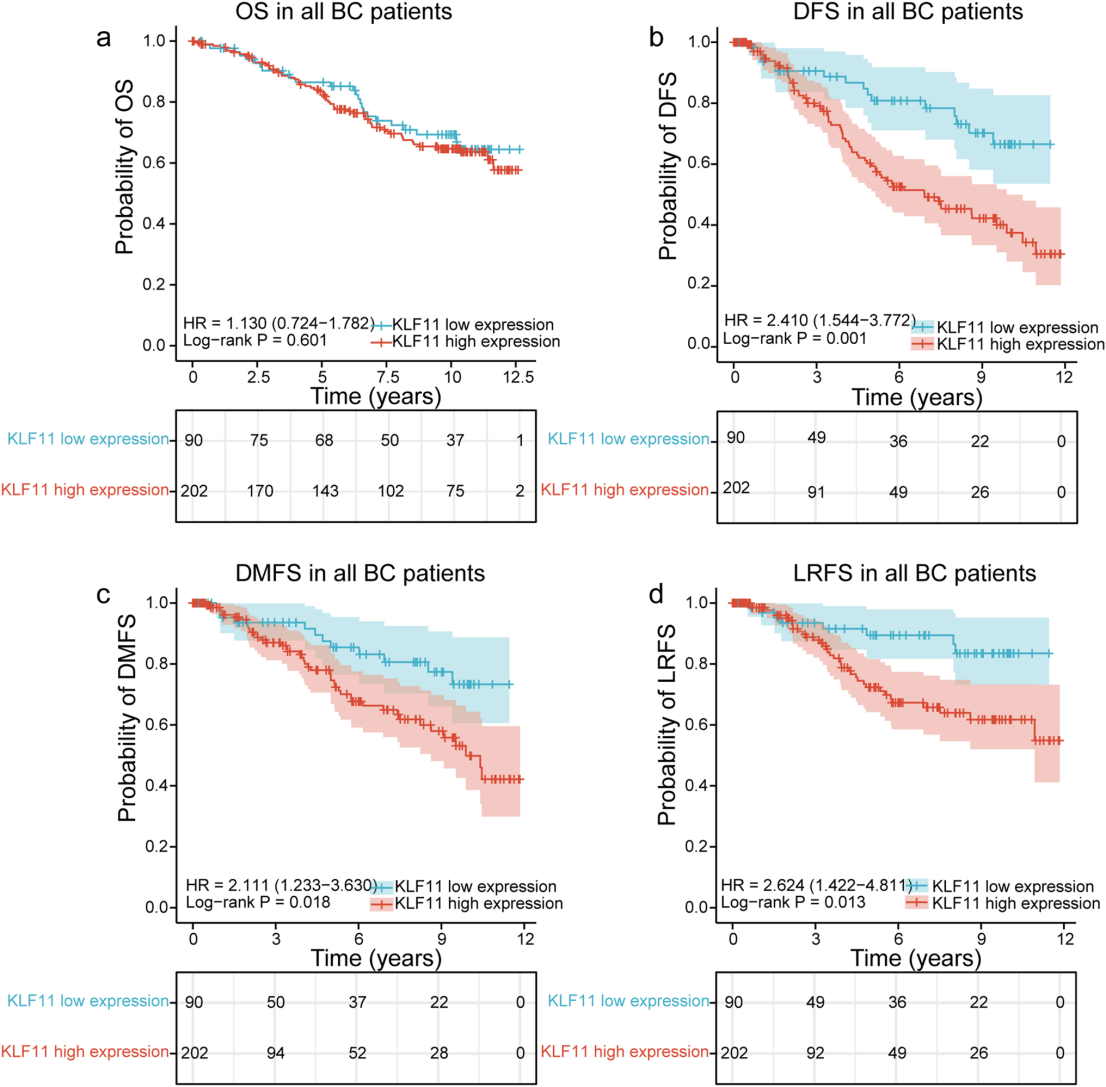
KLF11 was negatively associated with DFS, DMFS and LRFS in BC. **a** No prognostic significance of KLF11 for OS of BC patients was observed (HR = 1.130, 95% CI = 0.724–1.782, log-rank *p* = 0.601, **a**). **b-c.** Patients with categorized as high KLF11 expression showed poor DFS (HR = 2.410, 95% CI = 1.544–3.772, log-rank *p* = 0.001, **b**), DMFS (HR = 2.111, 95% CI = 1.233–3.630, log-rank *p* = 0.018, **c**) and LRFS (HR = 2.624, 95% CI = 1.422–4.811, log-rank *p* = 0.013, **d**). Kaplan–Meier analysis was performed and log-rank p value was calculated. *KLF11* Krüppel like Factor 11, *BC* Breast cancer, *OS* Overall survival, *DFS* Disease-free survival, *DMFS* Distant-metastasis-free survival, *LRFS* Local recurrence-free survival, *HR* Hazard ratio, *CI* Confidence interval

Of the overall patient cohort, patients categorized as high KLF11 expression showed poor DFS (HR = 2.410, 95% CI = 1.544–3.772, *p* = 0.001, Fig. 2b), DMFS (HR = 2.111, 95% CI = 1.233–3.630, *p* = 0.018, Fig. 2c) and LRFS (HR = 2.624, 95% CI = 1.422–4.811, *p* = 0.013, Fig. 2d). However, no association of KLF11 with OS of BC patients was found (HR = 1.130, 95% CI = 0.724–1.782, *p* = 0.601, Fig. 2a).

In addition, we explored the prognostic role of KLF11 in subgroups that grouped by the clinicopathological characteristics. Concerning OS, no prognostic significance of KLF11 was found (Supplementary Fig. 1), which was consistent with the survival analysis across overall-BC cohort. Interestingly, the subgroup survival analysis of DFS (Supplementary Fig. 2) showed that higher expression of KLF11 led to an inferior DFS both in patients younger than 50 years old (yo) (HR = 5.021, 95% CI = 1.174–21.477, *n* = 72, *p* = 0.030) and patients older than 50 yo (HR = 2.016, 95% CI = 1.103–3.686, *n* = 220, *p* = 0.023). KLF11 was negatively associated with DFS both in BC patients with the appearance of unifocal tumors (HR = 2.487, 95% CI = 1.090–5.672, *n* = 157, *p* = 0.034) and multifocal/multicentric tumors (HR = 2.505, 95% CI = 1.201–5.221, *n* = 135, *p* = 0.014). However, a higher expression of KLF11 led to shorter DFS of the patients only with histological type of invasive ductal carcinoma and/or invasive lobular carcinoma (HR = 2.173, 95% CI = 1.247–3.787, *n* = 238, *p* = 0.006), of Luminal A-like BC (HR = 2.831, 95% CI = 1.341–5.978, *n* = 163, *p* = 0.006), of patients with G1 or G2 BC ( HR = 3.108, 95% CI = 1.075–8.976, *n* = 117, *p* = 0.036), of patients without lymphatic metastasis (HR = 4.255, 95% CI = 1.650–10.976, *n* = 161, *p* = 0.003), of patients with tumor size ≤ 2 cm (HR = 3.268, 95% CI = 1.517–7.028, *n* = 190 *p* = 0.002), of patients with ER positive (HR = 2.740, 95% CI = 1.488–5.045, *n* = 236 *p* = 0.001), of patients with PR positive (HR = 2.591, 95% CI = 1.259–5.334, *n* = 169 *p* = 0.012) and of patients with HER2 unamplified statues (HR = 2.414, 95% CI = 1.343–4.339, *n* = 259, *p* = 0.003) and of the BC subgroup with a low proliferation rate (Ki-67 ≤ 14%) (HR = 2.831, 95% CI = 1.341–5.978, *n* = 164, *p* = 0.006).

Moreover, regarding the DMFS (Supplementary Fig. 3) subgroup survival analysis across clinicopathological subgroups, KLF11 was negatively correlated to DMFS only of the BC patients with histological type of invasive ductal carcinoma and/or invasive lobular carcinoma (HR = 1.926, 95% CI = 1.008–3.683, *n* = 238, *p* = 0.047), of patients without lymphatic metastasis (HR = 6.256, 95% CI = 1.451–26.962, *n* = 161, *p* = 0.014), of patients with tumor size ≤ 2 cm (HR = 2.690, 95% CI = 1.004–7.205, *n* = 190, *p* = 0.049), of patients with ER positive (HR = 2.308, 95% CI = 1.097–4.853, *n* = 236, *p* = 0.028), of patients with PR positive (HR = 2.751, 95% CI = 1.047–7.228, *n* = 169, *p* = 0.041) and of patients with HER2 unamplified statues (HR = 2.169, 95% CI = 1.076–4.374, *n* = 259, *p* = 0.033).

Furthermore, the subgroup survival analysis of LRFS (Supplementary Fig. 4) showed that high KLF11 expression correlated to impaired LRFS only of patients that aged older than 50 yo (HR = 2.382, 95% CI = 1.032–5.493, *n* = 220, *p* = 0.042), of patients with histological type of invasive ductal carcinoma and/or invasive lobular carcinoma (HR = 2.297, 95% CI = 1.054–5.005, *n* = 238, *p* = 0.036), of Luminal A-like BC (HR = 3.261, 95% CI = 1.220–8.722, *n* = 163, *p* = 0.019), of patients with the appearance of unifocal tumors (HR = 3.001, 95% CI = 1.028–8.761, *n* = 157, *p* = 0.044), of patients without lymphatic metastasis (HR = 3.946, 95% CI = 1.157–13.463, *n* = 161, *p* = 0.028), of patients with tumor size ≤ 2 cm (HR = 4.329, 95% CI = 1.504–12.461, *n* = 190, *p* = 0.007), of patients with ER positive (HR = 3.651, 95% CI = 1.522–8.756, *n* = 236, *p* = 0.004), of patients with PR positive (HR = 2.662, 95% CI = 1.017–6.968, *n* = 169, *p* = 0.046) and of patients with HER2 unamplified statues (HR = 2.627, 95% CI = 1.156–5.970, *n* = 259, *p* = 0.021) and also of the BC subgroup with a low proliferation rate (Ki-67 ≤ 14%) (HR = 3.261, 95% CI = 1.220–8.722, *n* = 164, *p* = 0.019).

In summary, combined the survival analysis, we could demonstrate that KLF11 was negatively associated with DFS, DMFS and LRFS in BC. However, the prognostic significance of KLF11 might be better correlated with outcome in some BC subgroups that characterized with less aggressive features.

### KLF11 was an independent prognostic factor for poor DFS and DMFS in BC

Next, we performed cox regression analysis to test independent prognostic potential of KLF11 in BC. Univariate cox regression analysis showed that KLF11 (HR = 2.433, 95% CI = 1.407–4.208, *p* = 0.001), grading (HR = 1.940, 95% CI = 1.106–3.403, *p* = 0.021), tumor size (HR = 1.991, 95% CI = 1.301–3.047, *p* = 0.002) and lymph node status (HR = 1.832, 95% CI = 1.187–2.829, *p* = 0.006) were significantly associated with DFS across the BC cohort (Table 2). Multivariate cox regression analysis was further applied with the univariate significant factors: grading, tumor size, lymph node status and KLF11 expression. The result revealed that KLF11 (HR = 2.610, 95% CI = 1.241–5.488, *p* = 0.011), grading (HR = 2.260, 95% CI = 1.262—4.047, *p* = 0.006) and tumor size (HR = 2.624, 95% CI = 1.384 − 4.975, *p* = 0.003) were independent factors for poor DFS (Table 2).

**Table 2:** Univariate and multivariate Cox regression analyses of KLF11 and clinicopathological characteristics for DFS in BC patients

Characteristics	Univariate analysis			Multivariate analysis		
	*p*	HR	95% CI	*p*	HR	95% CI
Age(< 50 yo vs. ≥ 50 yo)	0.223	1.337	0.893–2.130	n.i	n.i	n.i
Molecular subtype (non-LuA like vs.LuA-like)	0.137	1.379	0.903–2.106	n.i	n.i	n.i
Tumor histology (Invasive Ductal & Invasive lobular & Mixed type vs. other types)	0.075	1.938	0.935–4.015	n.i	n.i	n.i
Grading (G3 vs. G1-G2)	0.021*	**1.940**	**1.106–3.403**	0.006**	**2.260**	**1.262–4.047**
Tumor focis (multifocal & muticentric vs.unifocal)	0.238	1.292	0.844–1.976	n.i	n.i	n.i
Tumer size (pT2-pT4 vs. pT1)	0.002**	**1.991**	**1.301–3.047**	0.003**	**2.624**	**1.384–4.975**
Axillary lymph node status (yes vs. no)	0.006**	**1.832**	**1.187–2.829**	0.901	1.042	0.547–1.985
ER status (ER + vs. ER-)	0.608	0.875	0.525–1.457	n.i	n.i	n.i
PR status (PR + vs. PR-)	0.404	1.202	0.780–1.852	n.i	n.i	n.i
HER2 status (HER2 + vs. HER2-)	0.544	1.208	0.565–2.226	n.i	n.i	n.i
Expression of Ki-67(Ki-67 > 14% vs. Ki-67 ≤ 14%)	0.091	1.576	0.931–2.671	n.i	n.i	n.i
KLF11 (High vs. Low)	0.001**	**2.433**	**1.407–4.208**	0.011*	**2.610**	**1.241–5.488**

Values in bold are statistically significant

*KLF11* Krüppel like Factor 11, *BC* Breast cancer, *yo* years old, *DFS* Disease-free survival; pT, pathological Tumor size, *ER* estrogen receptor, *PR* progesterone receptor, *HER2* human epidermal growth factor receptor 2, *LuA-like* luminal A-like, *non-LuA like* not luminal A-like, *HR* Hazard ratio, *CI* Confidence interval, *n.i.* not included in multivariate model, as *p* > 0.05 in univariate analysis; *p < 0.05; **p < 0.01; ***p < 0.001

Furthermore, regarding DMFS, univariate cox regression revealed that KLF11 (HR = 2 0.132, 95% CI = 1.125–4.043, *p* = 0.020), molecular subtype (HR = 1.772, 95% CI = 1.054–2.981, *p* = 0.031), grading (HR = 2.689, 95% CI = 1.281–5.644, *p* = 0.009), tumor size (HR = 3.044, 95% CI = 1.818–5.099, *p* < 0.001) and lymph node status (HR = 2.328, 95% CI = 1.355–3.998, *p* = 0.002) were significantly associated with DMFS in BC (Table 3). Multivariate cox analysis was then applied with the univariate significant factors: molecular subtype, grading, tumor size, lymph node status and KLF11. The result showed that KLF11 (HR = 2.744, 95% CI = 1.017–7.403, *p* = 0.046), grading (HR = 3.276, 95% CI = 1.424 – 7.536, p = 0.005), and tumor size (HR = 5.729, 95% CI = 2.266 − 14.484, *p* < 0.001) were independent factors for poor DMFS (Table 3). Concerning LRFS, KLF11 was not an independent prognostic factor (data not shown).

**Table 3:** Univariate and multivariate Cox regression analyses of KLF11 and clinicopathological characteristics for DMFS in BC patients

Characteristics	Univariate analysis			Multivariate analysis		
	*p*	HR	95% CI	*p*	HR	95% CI
Age(< 50yo vs. ≥ 50yo)	0.514	1.211	0.682–2.150	n.i	n.i	n.i
Molecular subtype (non-LuA like vs.LuA-like)	0.031*	**1.772**	**1.054–2.981**	0.367	1.488	0.628–3.529
Tumor histology (Invasive Ductal & Invasive lobular & Mixed type vs. other types)	0.100	2.158	0.862–5.400	n.i	n.i	n.i
Grading (G3 vs. G1-G2)	0.009**	**2.689**	**1.281–5.644**	0.005**	**3.276**	**1.424–7.536**
Tumor focis (multifocal & muticentric vs.unifocal)	0.074	1.607	0.955–2.704	n.i	n.i	n.i
Tumer size (pT2-pT4 vs. pT1)	< 0.001***	**3.044**	**1.818–5.099**	< 0.001***	**5.729**	**2.266–14.484**
Axillary lymph node status (yes vs. no)	0.002**	**2.328**	**1.355–3.998**	0.942	1 .035	0.415–2.579
ER status (ER + vs. ER-)	0.101	0.624	0.355–1.096	n.i	n.i	n.i
PR status (PR + vs. PR-)	0.479	0.831	0.497–1.388	n.i	n.i	n.i
HER2 status (HER2 + vs. HER2-)	0.182	1.565	0.811–3.108	n.i	n.i	n.i
Expression of Ki-67(Ki-67 > 14% vs. Ki-67 ≤ 14%)	0.083	1.796	0.927–3.480	n.i	n.i	n.i
KLF11 (High vs. Low)	0.020*	**2.132**	**1.125–4.043**	0.046*	**2.744**	**1.017–7.403**

Values in bold are statistically significant

*KLF11* Krüppel like Factor 11, *DMFS* Distant metastasis-free survival, *pT* pathological Tumor size, *non-LuA-like* not luminal A-like, *ER* estrogen receptor, *PR* progesterone receptor, *HER2* human epidermal growth factor receptor 2, *LuA-like* luminal A-like, *HR* hazard ratio, *CI* confidence interval, *n.i.* not included in multivariate model, as *p* > 0.05 in univariate analysis; *p < 0.05; **p < 0.01; ***p < 0.001

In summary, we found that KLF11 was negatively associated with DFS, DMFS and LRFS in BC. Furthermore, KLF11 remained to be an independent prognostic factor for poor DFS and DMFS of BC.

### KLF11-related prognostic model for DFS and DMFS showed high accuracy in predicting survival probability of BC

**Fig. 3:**
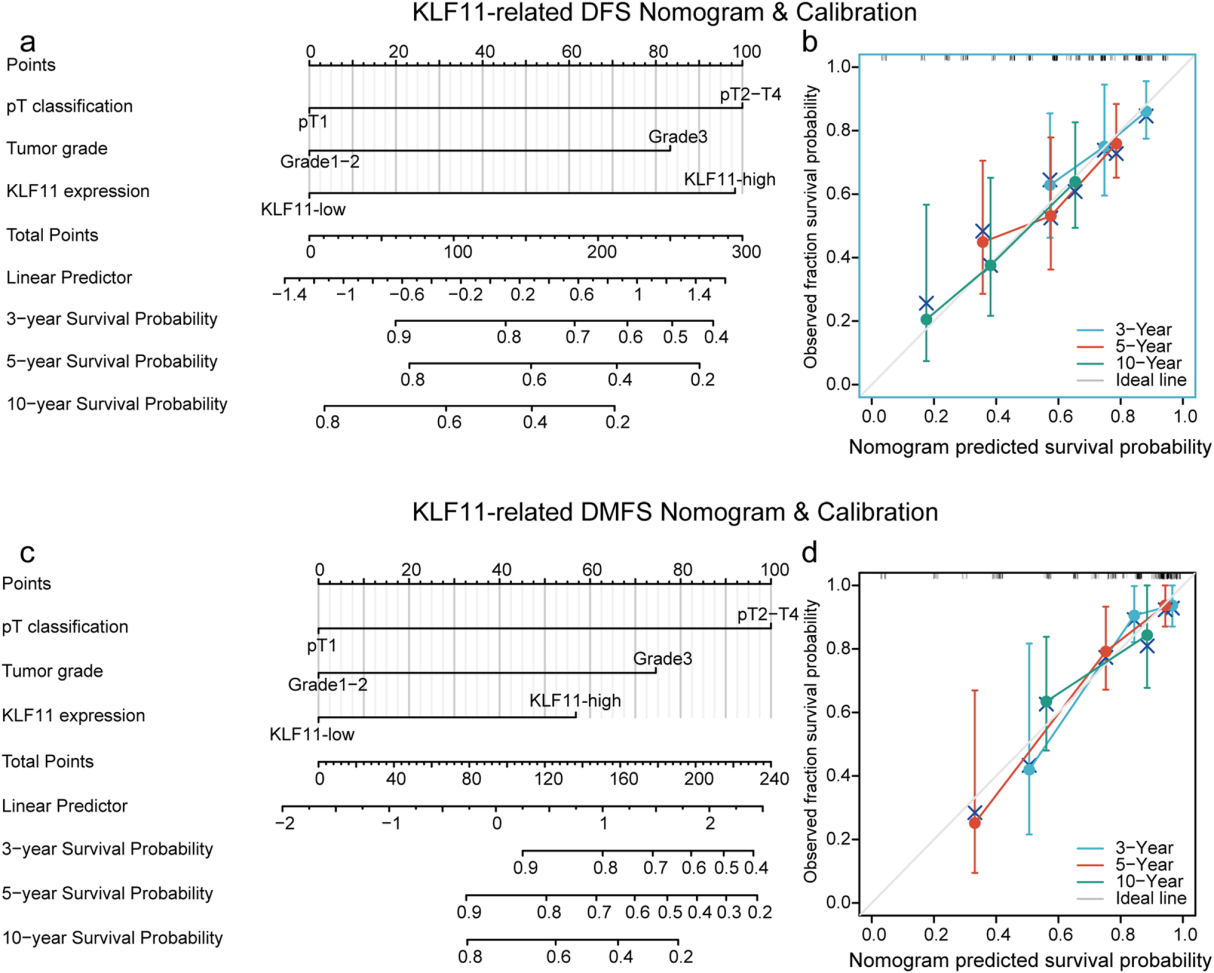
KLF11-related prognostic model for DFS and DMFS showed high accuracy in predicting survival probability of BC. **a**, **c** KLF11-related prognostic model displayed by nomograms for DFS (**a**) and DMFS (**c**) survival probability prediction of BC patients. Parameters that were independent predictors in the Cox regression models were used to develop the nomograms. Nomograms estimate the survival probability after 3, 5, and 10 years based on a total score calculated by the addition of zero to 100 points for every individual prognostic factor. For every parameter, a score on the upper points scale is given. For each patient, we calculated the points of the corresponding clinicopathological features and summed up all separate parameter points. The 3-year, 5-year, and 10-year survival probability can be estimated by drawing a vertical line from the "Total points" scale. **b**, **d.** Calibration curves showed the accuracy of the nomograms for predicting 3-year, 5-year, and 10-year DFS (**b**) and DMFS (**d**) survival probability. Nomogram-predicted survival is plotted on the x-axis, and actual survival is plotted on the y-axis. Vertical bars represent 95% CI measured by Kaplan–Meier analysis. Dashed lines along the 45° line through the origin point represent a perfect calibration model. *KLF11* Krüppel like Factor 11, *BC* Breast cancer, *DFS* Disease-free survival, *DMFS* Distant metastasis-free survival, *pT* pathological Tumor size

Based on the independent factors (including KLF11) revealed by the multivariate cox regression analyses, we developed two prognostic model displayed by nomograms in predicting DFS and DMFS for BC patients (Fig. 3). In the nomogram for predicting DFS (Fig. 3a), the KLF11 expression showed a high impact on the survival probability prediction due to the high level of KLF11 almost added up to 100 points to the final score. However, regarding DMFS, KLF11 expression only showed a minor impact on outcome prediction (Fig. 3c). The internal validation of the underlying regression models showed optimism adjusted c-index values of 0.694 for DFS and 0.8 for DMFS, respectively. The calibration of the prognostic models was assessed with calibration curves, which analyze the fit between the model established by the cox regression and the actual situation. The calibration curve of the nomogram-predicted DFS (Fig. 3b) showed that the predicted survival probabilities of the prognostic model for 3-, 5- 10- years were close to observed probabilities. The prediction accuracy for the 10-year DFS survival probability showed perfect match with the observed one estimated by the KM method (Fig. 3b). The predicted 3-, 5- and 10-year DMFS survival probability (Fig. 3d) also showed well fit with the actual DMFS of BC patients.

In summary, based on the multivariate cox regression analysis of the BC cohort, the constructed KLF11-related prognostic models for DFS and DMFS have a high accuracy in predicting the 3-,5- and 10 -year survival probability of BC patients.

### KLF11 induced proliferation and inhibited apoptosis in vitro

Taken together all the results from the cohort study, we could speculate that KLF11 might act as an oncogene in BC. To investigate the functional role of KLF11 in BC, we performed siRNA-mediated loss-of-function of KLF11 with cell viability, cell proliferation, and cell apoptosis assays in ER-positive luminal type (MCF7), HER2 amplified type (SK-BR-3), and TNBC (MDA-MB-231) cell lines. The successful knockdown was confirmed at the mRNA level by rtPCR (Supplementary Fig. 5a) and at the protein level by colorimetric cell-based KLF11 ELISA (Supplementary Fig. 5b).

**Fig. 4:**
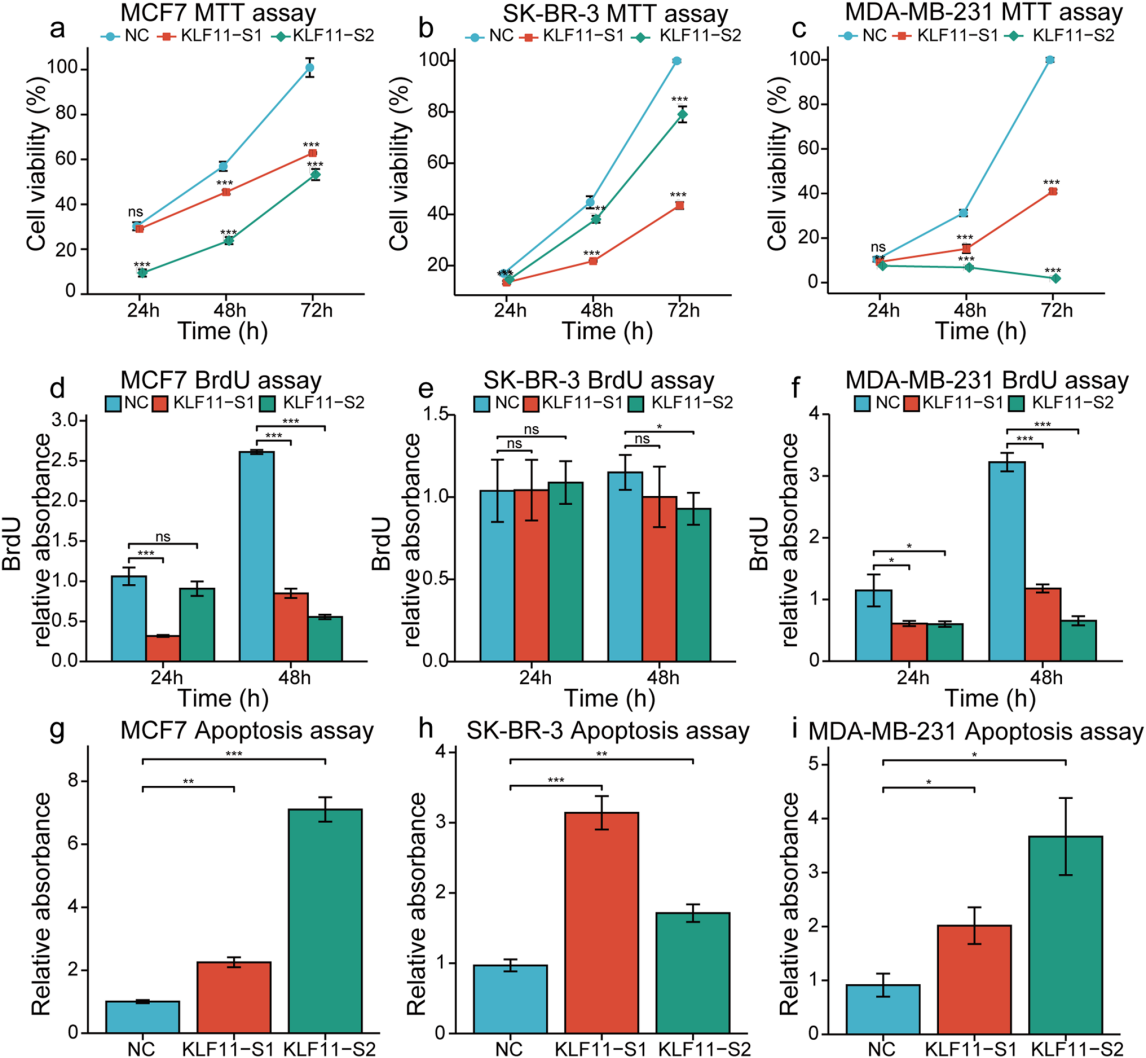
The knockdown of KLF11 inhibits cell viability and proliferation, as well as induced apop-tosis in BC cell lines. **a–c** MTT assay showed that the viable cells of MAF7 (**a**), SK-BR-3 (**b**), and MDA-MB-231 (**c**) were significantly reduced after transfection with KLF11-siRNAs compared to KLF11-NC. **d–f** BrdU assay showed the effect of cell proliferation of MCF7 (**d**), SK-BR-3 (**e**), and MDA-MB-231 (**f**) after transfection with KLF11-siRNAs compared to KLF11-NC. **g–i.** Apoptosis assay showed the effect of cell apoptosis of MCF7 (**g**), SK-BR-3 (**h**), and MDA-MB-231 (**i**) after transfection with KLF11-siRNAs compared to KLF11-NC. Error bars indicate the mean of ELISA triplicates in each experiment. All results are representative of at least three independent experiments. NC, negative control; One-way ANOVA test was performed to calculated the p values. ns, *p* > 0.05; *p < 0.05; **p < 0.01; ***p < 0.001

Then, the MTT assays showed that these three subtypes of BC cell lines showed a significant reduction of viable cells after transfection with *KLF11*-siRNAs (Fig. 4a–c). In addition, the highest inhibitory effect of KLF11 on cell viability was observed in the MDA-MB-231 cell line (Fig. 4c). Observing that the inhibition effect of *KLF11*-S1 and *KLF11*-S2 at the time point of 24 h of the MTT assay of MCF7 is inconsistent, additionally, we added the third siRNA (*KLF11*-S3) to further strengthen the credibility of the data for these three cell lines (Supplementary Fig. 5c). With the *KLF11*-S3 obtaining similar observation, these results indicated that KLF11 could promote BC cell growth, especially of TNBC. Moreover, the BrdU assays showed inhibition of proliferation after the knockdown of KLF11 in MCF7 (Fig. 4d) and MDA-MB-231 cells (Fig. 4f), which was not observed in SK-BR-3 cells. The inhibitory effect was more significant in MDA-MB-231 cells (Fig. 4f) than in MCF7 cells (Fig. 4d). These results indicated that KLF11 could inhibit cell proliferation only of MCF7 and MDA-MB-231 cell lines, especially of MDA-MB-232. In addition, the cell apoptosis assays showed that the knockdown of KLF11 induced apoptosis of MCF7 (Fig. 4g), SK-BR-3 (Fig. 4h), and MDA-MB-231 (Fig. 4i) cells. These results indicated that the downregulation of KLF11 could induce cell apoptosis of BC.

In summary, there was a high degree of consistency in cellular functional assays suggesting that KLF11 promotes tumor cell growth via promoting cell proliferation and/or suppressing cell apoptosis of BC. However, due to neoplastic intratumor heterogeneity, different molecular subtypes of BC are likely to have distinct underlying mechanisms.

## Discussion

The clinical BC cohort study demonstrated that KLF11 was positively associated with highly proliferative BC. Furthermore, BC patients with higher KLF11 expression led a shorter DFS, DMFS, and LRFS. KLF11 remained to be an independent prognostic factor for poor DFS and DMFS. The KLF11-related prognostic model for DFS and DMFS showed high accuracy in predicting the 3-,5- and 10 -year survival probability of BC patients. Furthermore, the subgroup survival analysis of KLF11 in BC patients grouped by clinicopathological parameters demonstrated that the prognostic significance of KLF11 might be correlated more closely with outcome in some BC subgroups that characterized with less aggressive features. In clinical practice, the more aggressive subtypes of BC often mean a lower survival probability and are treated with more aggressive therapeutic strategies. However, the less aggressive types of BC are often treated less aggressive. In fact, some of these patients will progress. It would be interesting for this group of patients to find an appropriate treatment schema. This prognostic profile of KLF11 has the potential to make it a screening molecular marker in less aggressive BC patients. Patients with relatively short survival and at risk of developing a more aggressive type could be selected by expression of KLF11. More aggressive treatment before their tumors progress would allow patients with such 'hidden' highly aggressive tumors to be treated more appropriately at the low aggressive stage and thus receive preventive measures before their outcome becoming worse.

Additionally, the in vitro functional assays in MCF7 and MDA-MB-232 cell lines demonstrated that KLF11 could promote cell viability and proliferation, and inhibited apoptosis of BC. However, in SK-BR-3 cell line, KLF11 only promoted cell viability and inhibited cell apoptosis without the observation of KLF11-promoted effect on cell proliferation. Additionally,, although the tendency of the impact of knockdown of KLF11 (by KLF11-S1 and KLF11-S2) on MCF7, SK-BR-3, and MDA-MB-232 cell lines was same, the induced extent of the cell apoptosis by different sequence of KLF11-siRNAs in different molecular subtype BC cell lines was shown to be different. This might be dependent on the different underlying mechanism of KLF11 involved in apoptosis in different subtypes of BC cell lines, which we also curious about. It is worth to noting that KLF transcription factor family is characterized by the possibility of performing different functions in different molecular subtypes of BC [11]. Since MCF7 and MDA-MB-231 cell lines are both HER2 negative type while SK-BR-3 is a HER2 positive type cell line, we speculate that the apoptotic pathways regulated by KLF11 in HER2 positive and HER2 negative BC cell line might be different [20]. It is not unexpected that the proteins transcriptionally regulated by KLF11 are also different. Considering the results of proliferation assay, the result of SK-BR-3 (HER2 positive) is negative, which is also totally different with MCF7 and MDA-MB-232 (HER2 negative) BC cell lines. We speculate that HER2 might be a vital factor for KLF11 exert its transcriptional regulation in BC. Different pathways are triggered and different protein populations were transcriptional regulated by KLF11 depends on different molecular subtype BC context, especially, the HER2 status. Our future study will be focus on the different underlying mechanisms of KLF11 in different subtype of BC cell lines to explore its possibility as an alternative and/or synergistic therapeutic targets for conventional targets (e.g., endocrine therapy or HER2-targeted therapy) for improving treatment of BC.

Interestingly, it has been demonstrated previously that miR-30d promotes BC cell growth, depending on the low expression of KLF11 [21]. This indicates that KLF11 might act as a tumor-suppressor in BC. However, present study directly demonstrated that KLF11 promoted BC cell growth via inducing cell proliferation and/or suppressing cell apoptosis of BC. Actually, in normal or untransformed cells, KLF11 does suppress cell proliferation and induce cell apoptosis, such as in pancreatic epithelial cells, normal ovary cells, etc., in vitro and in vivo [15, 22, 23, 24, 25, 26]. However, the role of KLF11 in tumor cell has shown to be reversed in several cancer types, including pancreatic cancer, ovarian cancer, etc. [15]. Mechanically, in normal or untransformed cells, KLF11 can strengthen TGFβ-induced cell growth inhibition and TGFβ-induced cell apoptosis [26, 27, 28]. However, this “strengthen route” is disrupted when the context turns to a tumor circumstance. For instance, in pancreatic cancer cells, due to the oncogene-RAS mutation, the KLF11-mSin3a interaction that as a vital part of the “strengthen route” is disrupted by the phosphorylation of KLF11 [27]. Consequently, KLF11 turns out to promote tumor growth [28]. Additionally, when the premalignant lesions develop into malignant tumor, the role of KLF11 has shown to be reversed due to the active of previously silenced oncogenic pathway. For example, KLF11 acts as a growth suppressor in Barrett’s epithelial cells by repression of prostaglandin E2 (PGE2) expression. During the carcinogenesis, due to the active of EGFR-AKT signaling pathway, this “repression route” is disrupted by the phosphorylated KLF11. Therefore, KLF11 turns out to be oncogene [29]. Intriguingly, Ras mutation and EGFR/AKT signaling activation also happen in BC. The underlying mechanism that reported for KLF11 in other types of cancer, whereby the role of KLF11 is reversed when the environment changes from a normal to a tumor situation, might also happens in BC. In-depth studies are necessary to explain and explore the regulatory mechanism of KLF11 in BC.

However, there are limitations. Our BC cohort is 20 years old (primary diagnosis 2000–2002). The advantage of an old patient cohort in terms of very long follow-up data is, in this case, also a disadvantage, as data on treatment cannot be obtained anymore. In addition, at that time, neoadjuvant chemotherapy therapies were not applied regularly in our clinic. However, the advantage of our data is that no patient in our cohort had undergone any prior treatment before surgery was performed. Regarding adjuvant therapy, even if we do not display it specifically, it is assumed to be the same in the two groups now sub-divided by KLF11-high/-low. Additionally, KLF11 was an independent prognostic factor in our cohort, which suggests that treatment (which depends on the other factors in this analysis, of which KLF11 were independent) should not cause any bias in our data. Since neoadjuvant chemotherapy has become common recently, this factor must be regarded in any research using more recent patient samples.

In conclusion, our study indicated that KLF11 might be a potential screening marker for patients with relatively short survival and at risk of developing a more aggressive type of in less aggressive molecular subtypes of BC. Most importantly, our study suggested that targeting KLF11 is an interesting therapeutic concept and further research could lead to a new therapeutic improvement in BC.

## Data Availability

The datasets generated and/or analyzed during the current study are available from the corresponding author on reasonable request.
